# Defining the Molecular Character of the Developing and Adult Kidney Podocyte

**DOI:** 10.1371/journal.pone.0024640

**Published:** 2011-09-08

**Authors:** Eric W. Brunskill, Kylie Georgas, Bree Rumballe, Melissa H. Little, S. Steven Potter

**Affiliations:** 1 Division of Developmental Biology, Cincinnati Children's Hospital Medical Center and the University of Cincinnati School of Medicine, Cincinnati, Ohio, United States of America; 2 Institute for Molecular Bioscience, The University of Queensland, St. Lucia, Australia; Pennington Biomedical Research Center, United States of America

## Abstract

**Background:**

The podocyte is a remarkable cell type, which encases the capillaries of the kidney glomerulus. Although mesodermal in origin it sends out axonal like projections that wrap around the capillaries. These extend yet finer projections, the foot processes, which interdigitate, leaving between them the slit diaphragms, through which the glomerular filtrate must pass. The podocytes are a subject of keen interest because of their key roles in kidney development and disease.

**Methodology/Principal Findings:**

In this report we identified and characterized a novel transgenic mouse line, MafB-GFP, which specifically marked the kidney podocytes from a very early stage of development. These mice were then used to facilitate the fluorescent activated cell sorting based purification of podocytes from embryos at E13.5 and E15.5, as well as adults. Microarrays were then used to globally define the gene expression states of podocytes at these different developmental stages. A remarkable picture emerged, identifying the multiple sets of genes that establish the neuronal, muscle, and phagocytic properties of podocytes. The complete combinatorial code of transcription factors that create the podocyte was characterized, and the global lists of growth factors and receptors they express were defined.

**Conclusions/Significance:**

The complete molecular character of the *in vivo* podocyte is established for the first time. The active molecular functions and biological processes further define their unique combination of features. The results provide a resource atlas of gene expression patterns of developing and adult podocytes that will help to guide further research of these incredible cells.

## Introduction

It has been stated that the kidney podocyte is a most spectacular cell type [1]. Podocytes exhibit a particularly striking shape, protruding multiple axonal like projections that surround the glomerular capillaries. Still smaller projections, the foot processes, extend further, delicately and precisely interdigitating, leaving between them the narrow slit diaphragms, through which the glomerular filtrate passes.

Podocytes have been shown to carry out many critically important functions. Along with the glomerular endothelial cells they synthesize the glomerular basement membrane [2]. The slit diaphragm, an extracellular extension of the podocyte, is the final filtration barrier, representing an important seal that prevents loss of proteins into the urine [1]. In addition podocytes function as pericytes, counteracting the distending forces of the high pressure perfusion of glomerular capillaries. The podocyte is also thought to play a key role in the constant cleaning of the GBM filter, required to prevent clogging [3].

During development podocytes are derived from the capping mesenchyme, which is induced by the ureteric bud [4]. The earliest differentiating podocytes are detected in the S-shaped bodies, where cells that abut the forming Bowman's space are seen to express podocyte specific markers, such as MafB. These early podocytes also synthesize Vegf, which initiates the recruitment of endothelial cells into the cleft. Podocytes are also thought to represent sites of initial injury for a number of important kidney diseases, including focal segmental glomerulosclerosis [5] and diabetic nephropathy [6]. Foot process effacement and podocyte loss are among the earliest detected cytologic changes in these diseases.

Because of its considerable medical importance the podocyte has been the subject of intense investigation. Of particular relevance to this report, a number of outstanding studies have examined the sets of genes that podocytes express. Some early pioneering work used RT-PCR coupled with laser capture microdissected podocytes, or podocytes harvested from freshly dissected glomeruli, to examine expression levels of restricted numbers of genes in biological samples [7], [8], [9]. Another important study constructed cDNA libraries and cDNA microarrays from isolated glomeruli, and identified podocyte enriched transcripts as well as genes altered in expression in *Foxc2* mutants [10]. Podocytes grown in culture have also contributed greatly to our understanding of podocyte biology [11], [12], [13]. Both cDNA and oligonucleotide microarrays have been used to examine changing global gene expression profiles of cultured podocytes exposed to mechanical stress [14] or high glucose levels [15]. Nevertheless, “many cellular functions change during culturing of cells, and therefore, results obtained from podocytes in culture need to be interpreted with care” [1].

In this report we used transgenic mice showing podocyte specific GFP expression to facilitate the rapid purification of podocytes from mice at gestational days E13.5 and E15.5, as well as adult. We then used microarrays to give global, sensitive and quantitative measures of podocyte gene expression at these different developmental stages. The resulting comprehensive definition of the podocyte gene expression state provides remarkable insight into the molecular character of this unique cell type. All of its expressed growth factors, receptors, and transcription factors are defined. Novel molecular markers of the podocyte are identified. In addition, the multifunctional features of this exceptional cell type are better characterized, identifying specific neuronal, phagocytic and muscle traits. This universal atlas of podocyte attributes represents a valuable resource to guide further studies of this fascinating cell.

## Results and Discussion

To more fully define the molecular character of *in vivo* podocytes we conducted a series of gene expression profiling experiments. The purpose was to globally define the changing gene expression states of this remarkable cell from stage E13.5 of development to adult. To this end we made use of the *MafB-GFP* BAC transgenic mouse from the GENSAT project [16]. We found that these mice showed highly restricted GFP expression in podocytes in both the developing and adult kidney.

**Figure 1:**
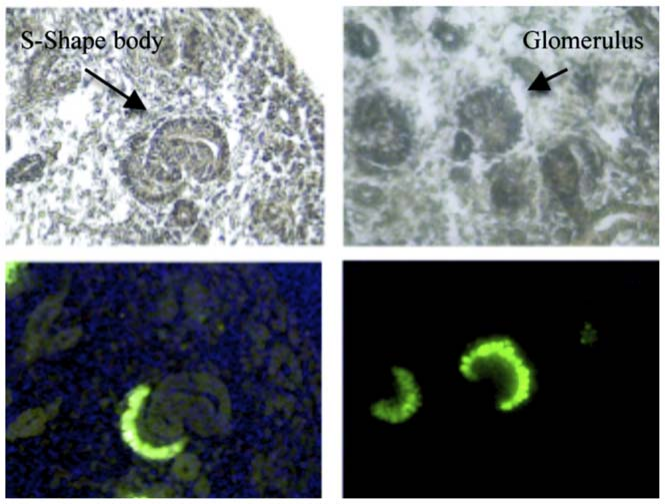
*MafB-GFP* mice show restricted expression of GFP in podocytes. Cryostat sections of E15.5 *MafB-GFP* transgenic kidneys with visible (above panels) and fluorescent (below) illumination. An S-shaped body and glomerulus are marked in above panels (arrows), and prospective podocytes in these structures are shown to be GFP positive in lower panels.

The podocyte specificity of *MafB-GFP* label was clearly demonstrated by fluorescent microscopy. Even as early as E13.5 in the S-shaped bodies the prospective podocytes were uniquely labeled by GFP (Fig. 1, left panels). At this stage of development the immature podocytes form a single layer of cells adjacent to the glomerular cleft. As development progresses a capillary loop forms within the cleft and the early glomerulus is encircled by podocytes (Fig. 1, right panels). The *MafB-GFP* transgenic kidneys did not show GFP fluorescence in cell types other than podocytes. In addition, the *MafB-GFP* fluorescence pattern was observed to exactly match expression patterns of known podocyte marker genes, as discussed in more detail later.

We used a strategy of rapid enzymatic cell dissociation followed by fluorescent activated cell sorting (FACS) to isolate the *MafB-GFP* positive cells from embryonic kidneys, at stages E13.5 and E15.5. For analysis of adult podocytes we first sieve purified glomeruli, which provided a significant enrichment for podocytes. The glomeruli were then further subjected to enzymatic dissociation and FACS, in order to obtain a pure population of podocytes. The *MafB-GFP* GFP fluorescent label was quite strong, allowing a stringent gating during FACS, which resulted in very low levels of contamination.

The resulting podocyte microarray data was further screened to monitor for possible contamination. One method used for estimating podocyte cell purity was to determine the transcript level for a marker of a flanking cell type. In particular, we examined expression levels for *Tie2* (*Tek*), which is specifically expressed in endothelial cells, which abut the podocyte and are therefore the most likely source of contamination. Samples with significantly above background levels of *Tie2* expression were removed from further analysis (see Material and Methods). In addition we have previously defined cell type and compartment level specific markers for most of the elements of the developing kidney (GUDMAP.ORG, [17]). The podocyte microarray data was carefully examined to insure against contamination by these multiple components.

### Defining the adult podocyte gene expression state

The podocyte has a unique multifunctional character that we wished to better define by examining its complete gene expression state. We used two strategies to screen the expression data. First, to discern the adult podocyte specific components we subtracted out the gene expression profile of the total adult kidney cortex. That is, we sought genes with transcripts specifically enriched in the podocytes compared to total cortex. To this end we used GeneSpring software, with summarization algorithm RMA16, filtered on raw expression minimum of 150 in at least three samples, performed ANOVA P<0.05, and required minimum three fold enrichment in adult podocytes compared to total cortex, giving a total of 436 probesets.

In a second strategy we searched for genes that were active in the adult podocyte compared to the developmentally early E13.5 podocyte. The underlying hypothesis is that the E13.5 podocyte is largely undifferentiated, and as the differentiation program ensues the set of genes that distinguish podocytes from other cells will become active. A similar analysis of the data was performed, requiring a minimum three fold enrichment, this time between adult podocytes and E13.5 podocytes. This approach yielded 739 probesets, with the two strategies having an overlapping set of 281 probesets.

Each strategy alone is imperfect. Some genes that are of key importance in the podocyte might also show significant expression in other regions of the kidney cortex. These genes would be unfortunately subtracted in the screen looking for enrichment compared to kidney cortex. And, some genes that are specific markers of the podocyte, such as *MafB*, are already active at E13.5. These genes would be eliminated by a screen that looks only for genes with transcripts enriched in the adult podocyte compared to E13.5. Nevertheless it is reassuring that the two strategies yield similar sets of genes, with about 2/3 of the 436 probesets found by comparison to kidney cortex also identified by the screen comparing adult to embryonic.

**Figure 2:**
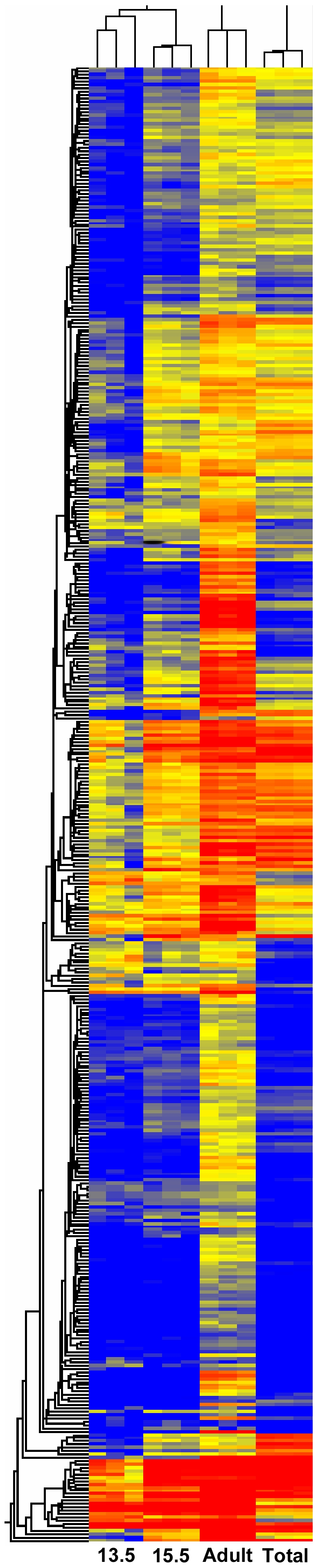
Heatmap of 894 probesets with elevated expression in adult podocytes. The 894 probset list was created by combining genes with three fold greater expression in adult podocytes compared to total adult kidney cortex (Total) or compared to E13.5 embryonic podocytes (13.5). Adult indicates adult podocytes, and 15.5 indicated E15.5 podocytes. Red marks strong expression levels, and blue shows weak expression. Note that for many probesets there is intermediate expression in E15.5 podocytes, compared to E13.5 and adult podocytes, suggesting that many podocyte specific marker genes show elevated expression as a function of developmental time. Most of the 894 probesets show increased expression in adult podocytes compared to both E13.5 podocytes and total kidney cortex, but some are only elevated relative to one or the other.

We first cast a wide net, looking to create a comprehensive catalog of genes whose expression defines the adult podocyte. We therefore combined the lists made with the two screening strategies creating a set of 894 probesets. A heatmap provides a visual representation of the relative abundances of transcripts of adult podocytes compared to E13.5, and E15.5 podocytes, as well as total kidney cortex (Fig. 2). Many of the genes show a graded expression level, weakest at E13.5, stronger at E15.5, and then strongest in the adult podocyte. Fig. 2 also illustrates how some of the adult podocyte probesets are enriched compared to E13.5 but not total cortex, while others are enriched versus total cortex but not E13.5. For a complete inventory of the 894 genes, along with fold enrichments, see Table S1. Of interest, and validating the screen, a large number of genes previously associated with podocytes showed the greatest enrichments. These results confirm the purity of the podocytes used for array analysis. For example, comparison of adult podocytes to total kidney cortex showed very strong fold changes (FC) for *Nphs1* (*nephrin*, 23 FC), *Nphs2* (*podocin*, 20 FC), *Wt1 (Wilm's Tumor1*, 19 FC), *Foxc2 (forkhead box c2*, 19 FC), *nes* (*nestin*, 12 FC) and *pdpn* (*podoplanin*, 17.5 FC) and *synpo* (*synaptopodin*, 7.5 FC) (Table S1).

Interestingly, we also found unexpected genes expressed in podocytes. For example *Foxd1*, which has generally been considered a marker of the kidney interstitium, or stromal lineage, showed extremely robust expression in the podocyte.

To better define the molecular processes and biological functions carried out by the podocyte we analyzed the 894 gene list with the ToppGene web tool [18]. This software application searches for gene enrichments associated with specific molecular functions and biological processes. An interesting view of the podocyte emerged, with an unusual mix of functions. Given the extraordinary structure of the podocyte it is not surprising that a number of enriched genes were associated with the cytoskeleton. There were 65 cytoskeletal binding proteins identified, and 39 genes involved in actin skeleton organization. Several other interesting molecular processes and biological functions emerged. Twelve genes encoded proteins involved in integrin binding, and another 44 were involved in calcium ion binding.

The top biological processes to emerge from the ToppGene analysis included vesicle mediated transport, with 72 genes involved, actin cytoskeleton organization (39 genes), regulation of signaling (99 genes), neurogenesis (74 genes), neuron projection development (52 genes), axon guidance (33 genes), biological adhesion (59 genes), response to oxygen levels (19 genes), neuromuscular junction (7 genes), chemotaxis (38 genes), phagocytosis (11 genes), striated muscle cell differentiation (16 genes), muscle contraction (20 genes). For complete gene lists see Table S2. The biological process analysis again shows a strong neuronal character, but also some molecular features associated with muscle and phagocytes.

We examined the possible muscle character of the podocytes in more depth, in light of their possible contractile role in counteracting the perfusion pressure of the capillaries. It is interesting to note that podocytes did express several myosins, including *myo6*, *myo1e*, *myo1d*, *myo10* and *myl6*. Nevertheless, these are generally unconventional myosins that are more associated with vesicle transport and other movements along actin filaments rather than muscle contraction. Podocytes also showed strong expression of *Tpm1*, tropomyosin, which binds actin filaments in both muscle and non-muscle cells.

It is interesting to compare the array results presented here with previous studies of the muscle nature of podocytes. Potential contractility of the podocyte has long been noted [19], and a more recent study examined in some detail the muscle characteristics of podocytes grown in culture [20]. Three muscle markers, smoothelin, calponin and myocardin were detected by three methods, microarray, western blot and immunofluorescence. Several other genes associated with smooth muscle differentiation were also seen elevated in expression in podocytes by as measured by microarray. Surprisingly, our microarray analysis of in vivo podocytes provided somewhat disparate results. We observed *Cnn1* (calponin) expression at essentially background levels in adult podocytes, although slightly above background at E13.5 and E15.5. *Myocd* (myocardin) was expressed only at background levels in podocytes for all times examined. Similarly *Smtn*, (Smoothelin), was just slightly above background (∼100 raw signal) at all times. In addition for several other muscle function genes previously observed expressed in podocytes [17], we saw little, if any, expression. *Neb*, *Rrad*, and *Ryr2* were at background levels, while *Fhl2*, *Adam8* and *Id1* were just slightly above background (∼100 raw signal). *Id2* showed moderate expression (∼400) in developing podocytes, but was off in adult, and *Aebp1* gave low level (∼250) adult expression. We did, however, observe very robust expression for both *Mbnl 1* and *2* (muslcebound like) in developing and adult podocytes. In summary we detected a distinct, yet rather weak, muscle gene expression signature. There are several possible reasons for the discrepancies. In particular, different microarrays were used, and the previous study primarily examined a podocyte cell line grown in tissue culture, compared to the *in vivo* podocytes isolated by FACS in this study.

### Podocyte Signaling

The gene expression profile analysis also provided a global view of the signaling pathways present in podocytes. A gene ontologies analysis of the 894 genes with podocyte elevated expression, using GeneSpring software, identified 44 genes in the receptor category, and 28 genes encoding proteins that bind receptors. These genes can be further screened by requiring a raw expression signal of at least 500, very roughly the transcription level required to be detected by *in situ* hybridization. This reduces the numbers to 26 receptor and 17 receptor binding genes. See Tables S3 and S4 for complete gene lists. Table S5 shows another list of 116 genes expressed in podocytes involved in signal transduction.

We found that podocytes express three semaphorins, *Sema3g*, *Sema5a* and *Sema3e*. Semaphorins are a large family of secreted and membrane bound proteins that act as axonal growth cone guidance molecules, primarily as short-range inhibitors, deflecting axons from inappropriate regions. It is easy to imagine how they could contribute to the formation of the slit diaphragm. It has previously been shown that immortalized podocytes in culture express semaphorins [21], [22], although many were reported expressed in near equal abundance, while we observed more restricted and distinct expression levels. It is also interesting to note that the *Sema3a* gene knockout mouse shows very wide foot processes and foot process effacement [22]. Our array data showed that *Sema3a* is expressed during kidney development, but is turned off in the adult podocyte. In contrast, *Sema3e* and *Sema3g* were off during development and expressed in the adult. *Sema5a* was expressed at low levels during development and strongly in the adult.

*Robo2*, another molecule with a key role in axon guidance, showed strong expression in adult podocytes. Of interest, one of the ligands, *Slit2*, showed modest expression in the podocyte at E15.5, but was off in the adult.

The *Npr3* gene, showed very robust expression, with a raw signal of around 4500 in the adult podocyte. This gene encodes a receptor for atrial natriuretic peptide, produced by the atria of the heart, with a number of vascular, renal and endocrine effects that are important in the maintenance of blood pressure and fluid volume. *Npr3* mutant mice show reduced ability to concentrate urine, and exhibit mild diuresis, as well as surprising bone defects [23]. Podocytes also expressed *Npr1*.

*Il6st* (*gp130*) encodes a protein that functions as part of receptors for IL6, LIF, OSM, CNTF, IL11 CTF1 and BSF3. We also observed strong expression of interferon gamma receptor 1 (*Ifngr1*) in adult podocytes. It has been previously observed that podocytes express this receptor and respond to gamma interferon with expression of HLA-DR, -DP and -DQ [24].

We also identified several integrin genes expressed by podocytes, including *Itga2*, *Itga3*, *Itgav*, *Itgb1* and *Itgb5*. ITGB1, heterodimerizes with ITGA3 forming a receptor for fibronectin, laminin, collagen, epiligrin, thrombospondin and CSPG4. Integrin alpha-V/beta-5 is a receptor for fibronectin, recognizing the sequence R-G-D.

The *Bmpr1a* receptor gene showed modest expression in the adult podocyte. It was previously reported that cultured mouse podocytes express *Bmpr1a*, *Bmpr1b*, *Bmpr2*, *Acvr1*, *Acvr2* and *Acvr2b*, as well as *Bmp2*, as determined using RT-PCR methods that could detect very low levels of expression [25]. Our array data shows only background levels of transcripts for *Bmpr1b*, and *Bmp2*, very low but microarray detectable expression levels for *Acvr1*, *Acvr2b*, modest expression for *Acvr2* and a high level of expression for *Bmpr2*, which is also expressed in embryonic podocytes, as well as the total kidney cortex.

Podocytes also expressed the *Mertk* gene, encoding a receptor kinase, which is of interest in terms of the possible clearing role of podocytes, since this gene has been shown to be important in the phagocytic function of some cell types [26]. Podocytes also expressed *Colec12a* scavenger receptor that has been shown to be important in the mediation of zymosan phagocytosis by vascular endothelial cells [27]. It has also been implicated in the clearance of amyloid beta in Alzheimers disease [28].

Other signaling molecules of particular interest expressed by podocytes included *Spred2*, which is a sprouty related inhibitor of receptor tyrosine kinases, and *Gmfb*, glial maturation factor, beta, which causes differentiation of brain cells, and inhibits proliferation [29].

Several genes related to calcium-regulated events showed elevated expression in podocytes, including *Anxa5* and *Anxa1*, calcium dependent phospholipid binding proteins implicated in exocytic and endocytic pathways. S100a10, is also calcium binding and also implicated in endocytosis and exocytosis. *Fyn* encodes a receptor tyrosine kinase that helps regulate intracellular calcium levels, and plays an important role in the brain in regulating axon growth, axon guidance and neurite extension.

Of particular note, podocytes also expressed *Vegf*, *Ctgf*, *Egf* and *Npnt* (nephronectin). *Angptl2* is a member of the VEGF family, and also showed very strong expression in the adult podocyte. Podocytes also expressed *Efnb1* (ephrin B1), a membrane bound ligand for Ephrin related receptor tyrosine kinases, which has been implicated in the orientation of axons.

In summary, the receptor and receptor binding gene expression signature of podocytes provide an interesting picture, showing roles for calcium signaling, phagocytic/clearing function, and overall a striking neuron like character for these cells.

### Transcription factor signature of the adult podocyte

Transcription factors generally play key roles in defining the identities of cells. Directed transcription factor expression can be used in some cases to drive the differentiation of stem cells towards defined cell types [30], or to trans-differentiate one cell type into another [31]. Remarkably, this strategy can also be used to reprogram differentiated cells into the functional equivalent of embryonic stem cells [32]. We were, therefore, particularly interested in defining the transcription factor gene expression code of the podocyte.

GeneSpring mediated gene ontologies analysis of the 894 genes with podocyte elevated expression identified 54 genes in the transcription category (Table S6). We found 34 of these showed over 500 raw expression signal. Included were 14 genes with very high expression levels of over 1000. It is striking that three of these genes, *Foxc2*, *Wt1* and *Pod1*, have been previously shown to play a key role in kidney, and podocyte development. Indeed, *Foxc2* and *Wt1*, coupled with early Notch signaling, have been shown to be capable of driving a podocyte development program [33].

*Foxd1*, another highly expressed gene in the podocyte is a member of the forkhead family of transcription factors, like *Foxc2*. Raw signal expression levels were observed to be over one thousand during development, at E13.5 and E15.5, and approximately 2,500 in the adult podocyte. *Foxd1* is generally considered a marker of stromal, or interstitial cells. The dramatic *Foxd1* mutant kidney phenotype, including reduced branching morphogenesis and decreased nephron numbers, has been attributed to a stromal cell function deficit in signaling to forming nephrons [34] or in formation of the renal capsule [35]. The robust expression of *Foxd1* observed in the podocyte, however, suggests a possible function mediated through this cell type. This result reasons that previous studies investigating *Foxd1* function in kidney development might be subject to re-interpretation. A podoctye specific knockout of *Foxd1* would be useful to determine its function in this cell type.

*Dach1*, like *Foxd1*, is highly expressed in the adult podocyte, with transcripts showing an approximate ten fold enrichment compared to total kidney cortex. It is also more widely expressed in the earlier developing kidney, but again including definite podocyte expression. The *Dach1* mutant mice exhibit early postnatal death, although no developmental defects were detected in any organ system examined, including kidneys [36]. The *Eya*, *Six* and *Dach* encoded proteins often interact in a conserved network in development [37]. We observed only background levels of *Eya1* and *Eya2* transcripts in the podocyte, and only slightly above background expression for *Eya3* and *Eya4*. Similarly, *Six1*, *3*, *4*, *5* and *6* were present at very low levels in the podocyte, while *Six2* transcripts were present at modest levels in the early developing podocyte, and essentially absent in the adult. *Dach1* in the podocyte therefore appears to act independent of *Six* and *Eya*. Of particular interest, given the importance of preventing adult podocyte proliferation [1], DACH1 can inhibit Cyclin D1 and thereby inhibit cell proliferation [38]. DACH1 can also inhibit JUN-mediated contact-independent growth [39], which could be of importance since we observe that *Jun* is also expressed in prodocytes. JUN is a positive regulator of cell proliferation. Of interest, loss of E-cadherin mediated cell-cell contact can up-regulate *Jun* [40].

Podocyte expressed transcription factors also included *Hoxc4*, *Hoxc6*, *Hoxc8*, *Zeb1*, and *Mafb*. Of interest, *Hoxc6* and *Hoxc8* are both expressed strongly in stromal cells during development, like *Foxd1*, as well as in forming podocytes (Genepaint). The targets of Hox genes can vary from cell type to cell type, but it is interesting to note that HOXC6 targets in prostate cells include elements of FGF, BMP, NOTCH and WNT signaling pathways [41], [42]. MAFB is a member of the Maf family of transcription factors, and has been previously shown to play a critical role in podocyte development, with mutant podocytes showing fused foot processes that did not interdigitate [42]. *Zeb1* encodes a zinc finger E-box binding homeobox transcription factor and has previously been associated with epithelial mesenchymal transition, in particular as a repressor of E-cadherin [43].

Other transcription related genes of interest strongly expressed in the podocyte included *Supt4h1*, a regulator of transcription pausing, *Sap18*, part of a histone deacetylases complex, *Smarca2*, a member of the SWI/SNF family of proteins that regulate gene expression through chromatin remodeling, *Setd7*, a histone methyltransferase, *Kdm4c* and *Jhdm1d*, both histone demethylases, *Txnip*, which can induce cell cycle arrest, and *Sync*, which is generally expressed in skeletal and cardiac muscle.

It is important to note the striking success of this screen in identifying a number of transcription factors previously shown to play key functional roles in the podocyte. This provides an important historical validation of the analysis. It suggests that some of the newly identified podocyte expressed genes, reported herein, may also have yet to be discovered essential functions in podocyte biology. It is also important to note that the analysis of podocyte transcription factor expression presented in this report is far from exhaustive. Many other transcription factors, as listed in supplementary data, that are not necessarily strongly enriched in the podocyte, and/or are not expressed at high levels, are certain to play important roles in the podocyte.

### Pathways analysis

**Figure 3:**
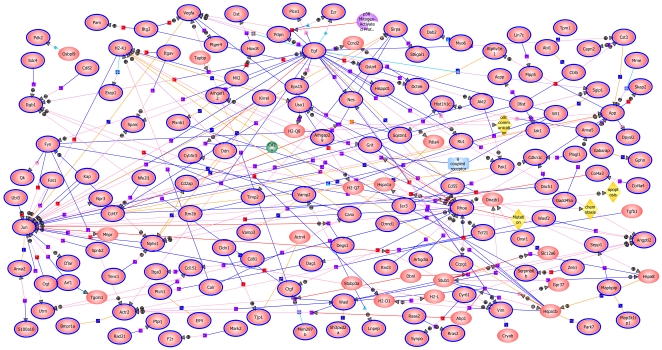
GeneSpring pathways analysis of the 894 genes with elevated podocyte expression. This diagram illustrates many of the known direct interactions between the proteins encoded by the 894 list of podocyte enriched genes. JUN, EGF, RHOA, as well as ITGB1, H2-K1, FYN, ACTR2, VEGFA, APP, ANGPTL2 and HSPA1B, show strong interaction centers. Ovals surrounded by blue lines represent proteins included in the 894 list.

A GeneSpring pathways analysis, looking for direct interactions, was carried out on the 894 podocyte probeset list (Fig. 3). The resulting diagram illustrates many of the known interactions among these gene products, and shows particularly strong interaction centers for EGF, JUN, and RHOA, as well as many interactions for ITGB1, H2-K1, FYN, ACTR2, VEGFA, APP, ANGPTL2 and HSPA1B. This begins to reveal the complex interplay of the podocyte specific gene products.

### Requiring greater podocyte stringency

The above analysis examined a broad set of podocyte enriched genes. There is, however, also utility in the identification of a more stringent set of podocyte expressed genes. This serves to further define the unique nature of the podocyte, provides a potentially valuable new set of molecular markers specific to the podocyte, and can serve to aid in the construction of novel transgenic tools for the study of the podocyte.

**Figure 4:**
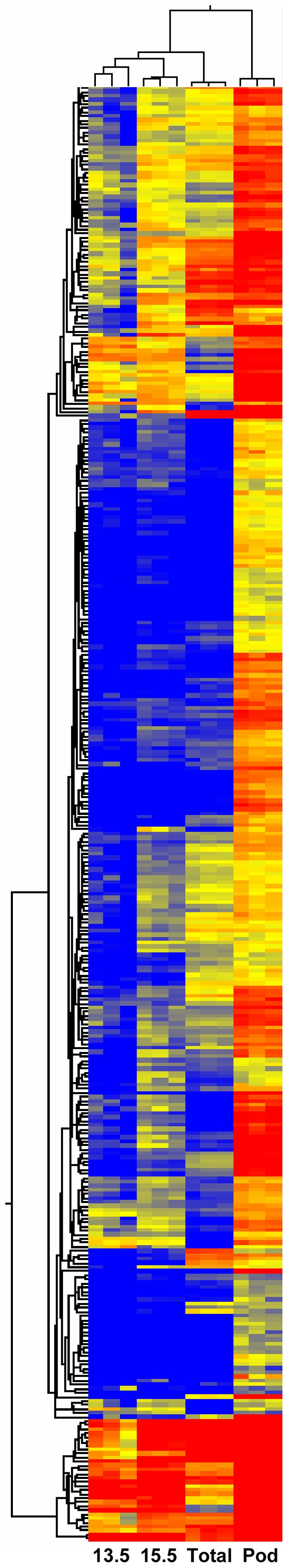
Heatmap of 365 probesets with five fold elevated expression in adult podocytes. A relatively stringent screen, requiring five fold enrichment in adult podocytes (Pod) compared to either total adult kidney cortex (Total), or E13.5 embryonic podocytes (13.5). Red indicates strong expression and blue weak expression.

**Figure 5:**
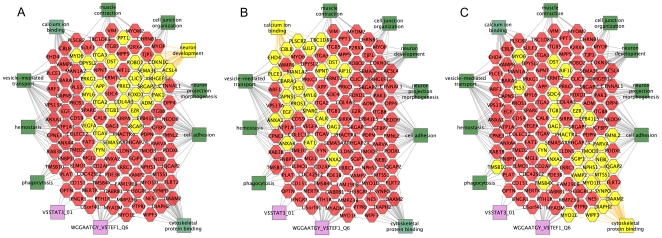
Functional groupings of genes with elevated adult podocyte expression. Cytoscape diagrams of a subset of genes from 365 probeset list of adult podocyte elevated genes (hexagons in center). Key biological processes and molecular functions (green), as well as upstream regulatory transcription factors (purple), are shown as surrounding rectangles, connected to associated genes by lines. A. Neuron related genes, B. Calcium binding related genes, and C. Cytoskeletal protein binding related genes, are highlighted in yellow.

To this end we repeated the analysis of the array data, only this time requiring five fold enrichment in podocytes, instead of the previous three. This resulted in 204 probesets for the podocytes versus total cortex comparison, and 304 probesets for the adult podocytes versus E13.5 podocyte comparison, with an overlap of 143 probesets (Fig. 4). The combined entity list of 365 probesets (Table S7) was then examined with ToppGene, finding similar sets of molecular functions and biological processes, compared to those identified with the 859 probeset list. These can be visually represented with cytoscape, with a subset of the 365 podocyte specific genes shown as hexagons in the center (Fig. 5). Associated molecular functions and biological processes are shown as green rectangles, linked by lines to related genes. Key functions identified include neuron development (Fig. 5A), calcium binding (Fig. 5 B), and cytoskeletal protein binding (Fig. 5 C). This functional analysis of genes lists is based on statistically significant gene enrichments. For example Table S2 shows that there are 72 podocyte genes associated with vesicle-mediated transport, and this is highly statistically significant, with a P value of zero, arguing very strongly that podocytes carry out vesicle mediated transport.

### Podocyte specific markers

**Figure 6:**
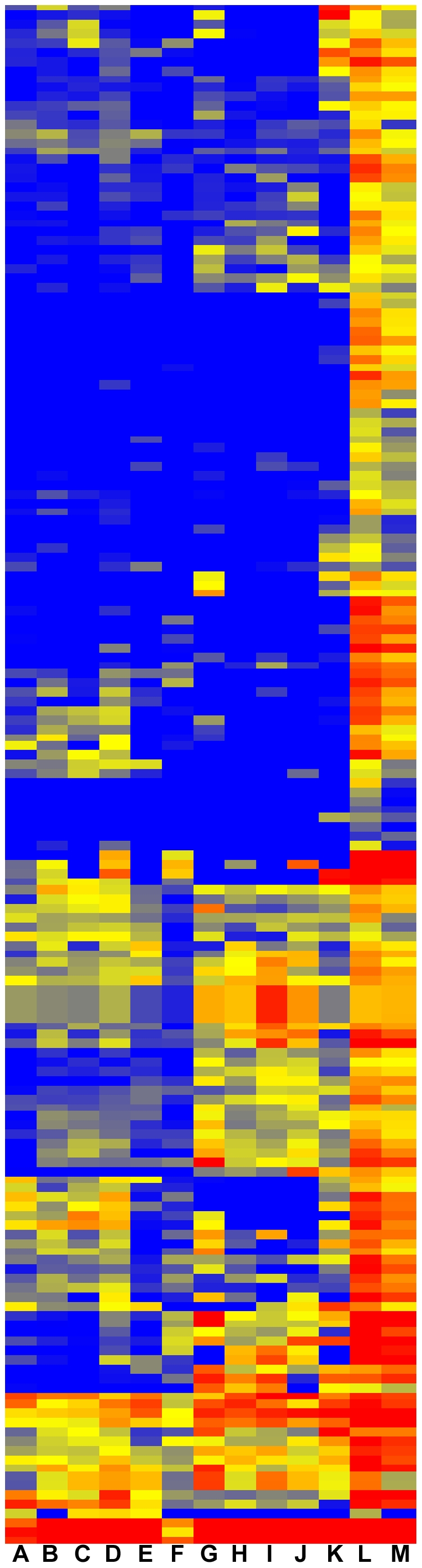
Comparison of podocyte expressed genes across many kidney cell types. Heatmap showing relative expression levels of most stringently selected 171 adult podocyte probesets. A. E13.5 podocytes. B. Renal vesicles from P4 mice. C. P1 Cap mesenchyme. D. E15.5 podocytes. E. E15.5 endothelial cells. F. Adult total kidney cortex. G. Adult *Meis1* expressing mesangial cells. H. Adult cortex endothelial cells. I. Adult glomerular endothelial cells. J. Adult medullary endothelial cells. K. Adult renal capsule. L. Adult podocytes. M. Adult total glomerulus.

We further extended the adult podocyte specific analysis, this time screening for genes with higher expression levels as well as five fold podocyte enrichment compared to total kidney cortex. In this case we did not include genes with only strong enrichment in adult compared to embryonic podocytes, as several of these genes were not podocyte specific. The heatmap of Fig. 6 shows the resulting 171 probeset list, with expression levels compared across 13 specific kidney compartments. A version of this heatmap with gene names included is provided in Figure S1. After removing probeset duplications and ESTs the list reduces to 144 podocyte highly enriched genes (Table S8).

**Figure 7:**
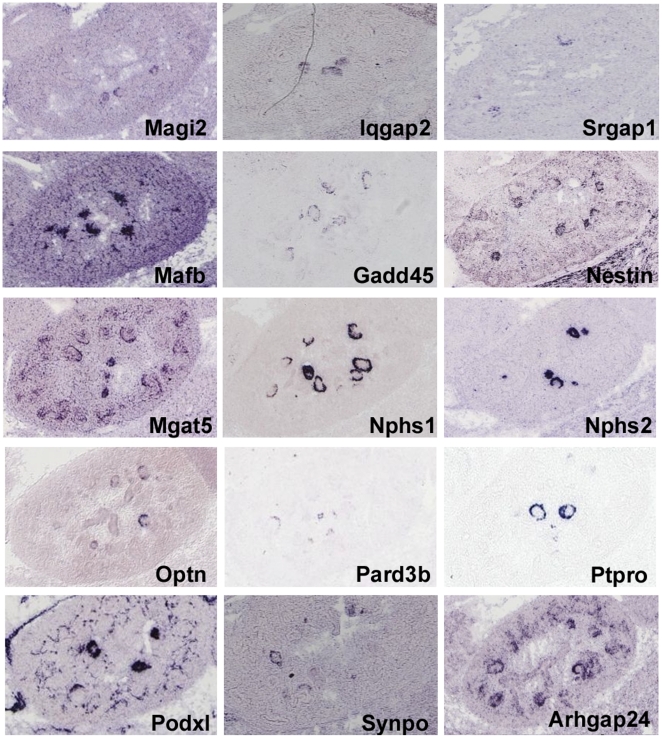
Expression of adult podocyte genes in the developing kidney. *In situ* hybridizations showing expression in the podocytes of E14.5 kidneys (GenePaint and Eurexpress). *Nphs1* and *Nphs2* serve as positive controls illustrating the circular or crescent appearance of podocyte specific expression, depending on the angle of the section.

**Figure 8:**
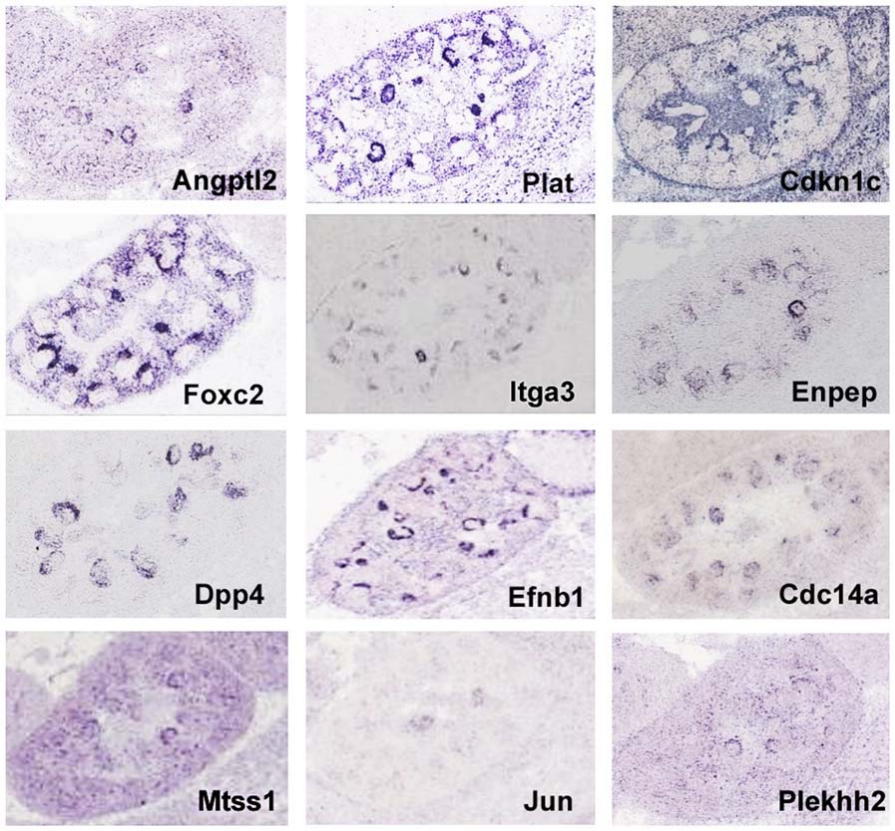
*In situ* hubridizations suggesting podocyte expression. Expression patterns for genes from the 144 list of genes with highly enriched expression in adult podocytes. For these genes the hybridization patterns suggest expression in even very early E14.5 podocytes. Data from GenePaint and Eurexpress.

These genes were then examined for podocyte expression using two public gene expression databases GenePaint (www.genepaint.org) and Eurexpress (www.eurexpress.org). These databases examine gene expression patterns by *in situ* hybridizations, using E14.5 mouse embryos. During these early stages of development the podocytes encase the developing glomerulus, resulting in distinctive crescent or circular expression patterns. The *Nphs*1 and *Nphs2* genes, which are known to be podocyte specific in expression, served as positive controls. The result was striking, with 30 genes showing hybridization patterns suggestive of podocyte expression (Fig. 7,8). This is a surprisingly large fraction of the 144 total, since the array data showed that many of the adult podocyte specific genes are not yet strongly expressed at early developmental stages (Fig. 2).

**Figure 9:**
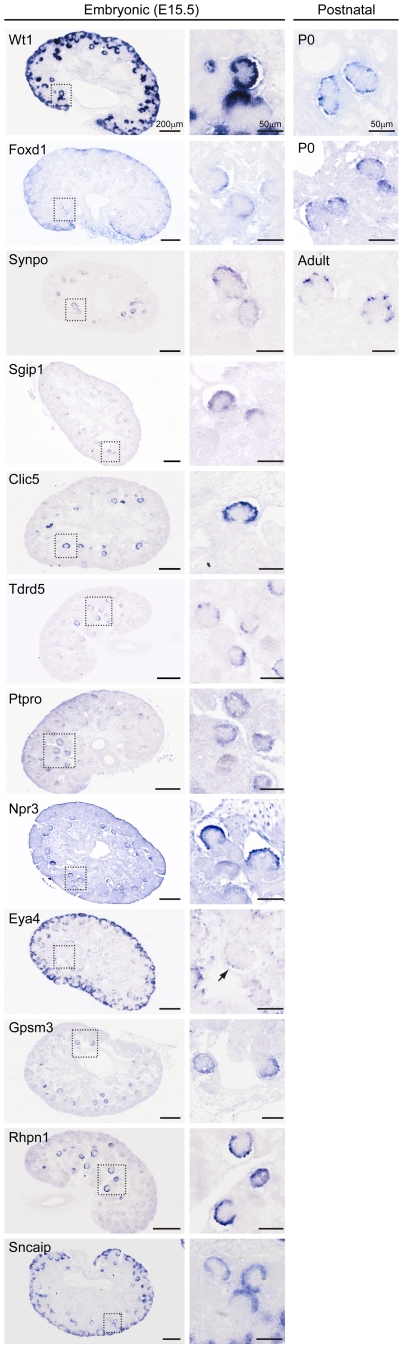
Expression of podocyte genes in E15.5 embryos, as well as P0 and adult. Genes expressed in podocytes of the embryonic (E15.5) and postnatal kidney (P0 or adult) detected by section *in situ* hybridization. The entire kidney is shown with a region enlarged to show podocyte expression in the renal corpuscle. For *Eya4*, in addition to the obvious strong cap mesenchyme expression, weak gene expression is detectable in podocytes (arrow).

To provide additional validation of the microarray data more *in situ* hybridizations were performed, at E15.5, and a third public database, GUDMAP.ORG, was used. The resulting gene expression patterns for twelve podocyte expressed genes are shown in Fig. 9. For some of these genes, such as *Wt1*, *Synpo* and *Clic5*, podoctye expression has been previously shown, It is important to note that they are expressed in the E15.5 kidney in cells that circle the capillary loop, exactly as observed for the *MafB-GFP* fluorescent cells. In addition this figure documents the podocyte expression of many additional genes. In some cases the expression is quite podocyte specific, as for *Rhpn1*, *Gpsm3*, *Tdrd5* and *Sgip1*, while in other cases there is also expression observed in other cell types. Of particular importance, the *in situ* hybridization results confirm the podocyte expression of *Foxd1*, which has previously been assumed to be a stromal cell specific marker.

### Summary

In summary we have carried out an analysis of the complete gene expression states of the podocyte during development and in the adult. The gene expression profiles provide a comprehensive definition of all of the transcription factors, growth factors, receptors and cytoskeletal proteins expressed by this remarkable cell type. The results confirm and extend previous studies. A distinctly neuronal character was defined for these cells, consistent with their axonal like projections, but seemingly contrary to their mesodermal origin. The podocytes also expressed a number of genes commonly associated with muscle, although when examined *in toto* their muscle gene expression signature was rather weak. Podocytes were also found to display phagocytic like properties, perhaps related to their function in the clearing of the GBM. In addition, an exhaustive list of podocyte expressed genes involved in calcium signaling were identified. This study, to the best of our knowledge, provides the most comprehensive analysis to date of the molecular character of this most spectacular cell type.

## Materials and Methods

### FACS purification of podocytes

*MafB-GFP* transgenic mice, Tg(Mafb-EGFP)FT79Gsat, were obtained from GENSAT/MMRC (http://www.gensat.org/MMRRC_report.jsp). Kidneys were dissected from E13.5 and E15.5 embryos and placed in ice cold PBS. Four kidneys from *MafB-GFP* transgenic mice, as identified with a fluorescence microscope, were transferred to a single 1.5 ml eppendorf tube and 400 µl of 0.05% Trypsin-EDTA was added, followed by incubation for 5 min at 37°C. The samples were triturated 30×, 600 µl of ice cold 0.1%BSA/PBS or 2% FBS/PBS was added, followed by an addition 30× trituration. Centrifuge 5 min 1500 rpm, 4°C, and discard supernatant. Resuspend in 600 µl of ice cold 0.1% BSA/PBS or 2% FBS/PBS, and centrifuge again at 1500 rpm, 4°C. Discard supernatant, resuspend cells in 600 µl 0.1% BSA/PBS or 2% FBS/PBS, filter using 70 µm mesh FACS collection tube, keep cells on ice, and immediately purify using a high speed digital BD FACS Aria II Cell Sorter.

For purification of podocytes from adult kidneys we used a similar strategy, only we first isolated glomeruli using a sieving method, allowing us to start with a dramatically enriched podocyte population. Kidneys were isolated in ice-cold PBS, bisected, and the medullary region was removed using a scalpel. The remaining cortical region was finely minced using a razor blade, placed into a solution of PBS/1.0% collagenase and incubated at 37°C for 15 min. The solution was vigorously triturated every five minutes. An equal volume of ice-cold 5% FBS/PBS was added and the mixture was filtered through a 100 µm mesh. The flow-through was collected, triturated vigorously and refiltered through a 100 µm mesh. This step was repeated twice. To collect the glomeruli, the flow-through was filtered using a 40 µm mesh. The glomeruli trapped in the filter were isolated by turning the filter over and rinsing with ice-cold 0.1% FBS/PBS. The collected glomeruli were vigorously triturated and re-filtered through 40 µM mesh. This process was repeated three times. After the final collection, the glomeruli were pelleted, washed with ice-cold PBS and resuspended in 0.5% trypsin, and incubated at 37°C for 10–15 min. During incubation, the glomeruli were triturated every five min. A portion of the sample was observed under a microscope to monitor cell dissociation. After incubation, ice-cold 5% FBS/PBS was added, the cells were pelleted, washed three times with ice-cold PBS and filtered before FACS analysis.

This study was carried out in strict accordance with the recommendations of the Guide for the Care and Use of Laboratory Animals of the National Institutes of Health. The protocol was approved by the Cincinnati Children's Research Foundation Institutional Animal Care and Use Committee (protocol number 0D02013).

### Purity

Podocyte purity was monitored by examining expression levels of genes established as molecular markers of endothelial cells, which represent the most likely source of contamination, since the podocyte resides opposite the glomerular endothelial cell. *Tie2* (*Tek*) is a marker of endothelial cells, where it typically gives raw signal levels of approximately 2,000 to 5,000. In our podocyte samples we typically observed *Tie2* expression levels below 100, or approximately background. For example, for six gene expression profiles generated for adult podocytes we observed *Tie2* raw expression signals of 60, 66, 64, 325, 504 and 1063. By this measure three of the samples showed significant endothelial contamination, and they were therefore deleted from the study. The podocyte microarray data was also examined for possible contamination from other cell types by looking at expression levels of other markers of multiple specific cells and compartments of the kidney, which we have previously defined [17].

The *MafB-GFP* transgenic mice gave very robust GFP signal during FACS purification, and stringent gating was used to reduce possible contamination levels (Figure S2).

### RNA purification and Target Amplification

RNA was purified using Qiagen RNeasy Micro Kits. Target amplifications were carried out using RiboSpia technology from Nugen, with the Ovation Pico WTA system. Affymetrix standard methods were used for carrying out microarray hybridizations, washes and scans.

### Array Data Analysis

Data was analyzed primarily using Agilent GeneSpring 11.5.1 software. Specific parameters are described in Results and Discussion. In addition we used ToppGene (http://toppgene.cchmc.org/) [18], ToppCluster (http://toppcluster.cchmc.org/) [44] and Cytoscape (http://www.cytoscape.org/) [45] for functional analysis and preparation of figures.

Microarray data is available on the public resources GUDMAP (www.gudmap.org), and GEO (GSE 17142, GSE17143, GSE17145).

### Section *in situ* Hybridisation

Section *in situ* hybridization was performed on paraformaldehyde-fixed embryonic (E15.5) or postnatal (P0 or adult) kidneys from CD1 mice. Digoxigenin-labeled riboprobes generated via PCR of E15.5 mouse cDNA (see Table S9 for primer sequences) were hybridized to paraffin-embedded and sectioned kidneys and detected via an alkaline phosphatase conjugated anti-Digoxigenin antibody (Roche 11093274910) and visualized using BM Purple color substrate (Roche 11442074001). The protocol has previously been described in detail [46], [47] and is available on the GUDMAP website via the Project Protocols page (http://www.gudmap.org/Research/Protocols/Little.html).

## Supporting Information

Figure S1 **Heatmap of genes with five fold enrichment in podocytes versus total cortex.** This corresponds to Fig. 6, only it includes gene symbols, which can be visualized by zooming in.
(JPG)

Figure S2 **FACS of cells from non-transgenic control (Panel A), and MafB-GFP transgenic (Panel B) mice.** The cells marked in green in Panel B are the GFP positive cells that were collected.
(TIF)

Table S1 **Combined list of 894 genes showing elevated podocyte expression.**
(XLS)

Table S2 **Functional analysis of 894 podocyte expressed genes.** Toppgene (http://toppgene.cchmc.org/), ToppFun was used to analyze the inclusive list of genes showing elevated expression in podocytes. Top molecular functions, biological processes, and cellular components, along with associated genes, are presented. In addition human phenotypes, mouse phenotypes, domains, pathways and diseases are listed. In addition the best candidate regulatory transcription factors, along with candidate target genes, as well as regulatory microRNAs and candidate target genes, are listed.
(XLS)

Table S3 **List of podocyte expressed genes associated with receptor activity.**
(XLS)

Table S4 **List of podocyte expressed genes associated with receptor binding.**
(XLS)

Table S5 **List of podocyte expressed genes associated with signal transduction.**
(XLS)

Table S6 **List of podocyte expressed genes associated with transcription.**
(XLS)

Table S7 **List of 365 genes with elevated podocyte expression.** This list is derived using a more stringent five fold enrichment, compared to the three fold enrichment used to make the 894 list.
(XLS)

Table S8 **List of 144 highly enriched, highly expressed podocyte genes.**
(XLS)

Table S9 **Table of primers used to make probes for in situ hybridizations.**
(XLS)
