# Supplementary figures and images for: Defining the Molecular Character of the Developing and Adult Kidney Podocyte
Source: PLoS One. 2011 Sep 8;6(9):e24640. doi: 10.1371/journal.pone.0024640 (PMC3169617; doi:10.1371/journal.pone.0024640)

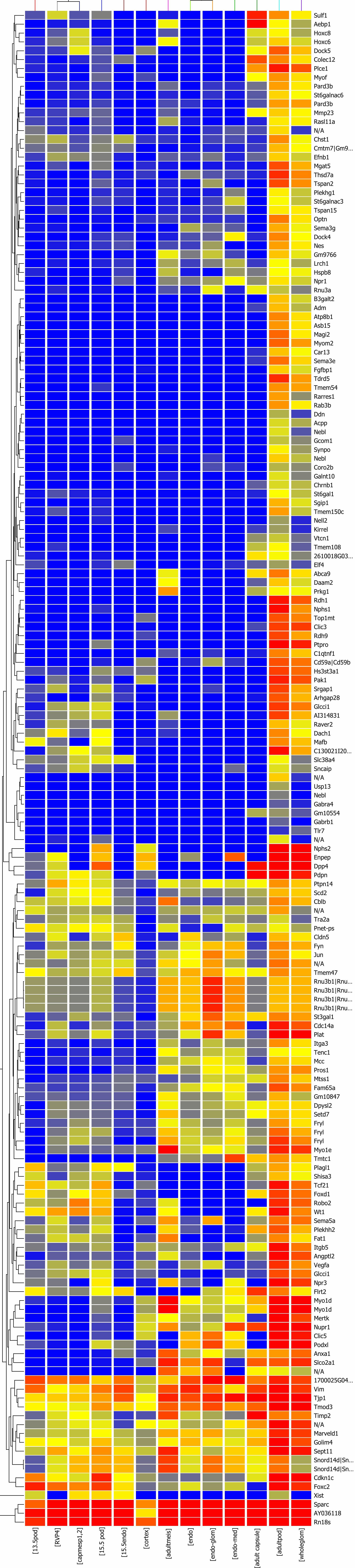

Supplement: Figure S1 — Heatmap of genes with five fold enrichment in podocytes versus total cortex. This corresponds to Fig. 6, only it includes gene symbols, which can be visualized by zooming in. (JPG) [file pone.0024640.s001.jpg]

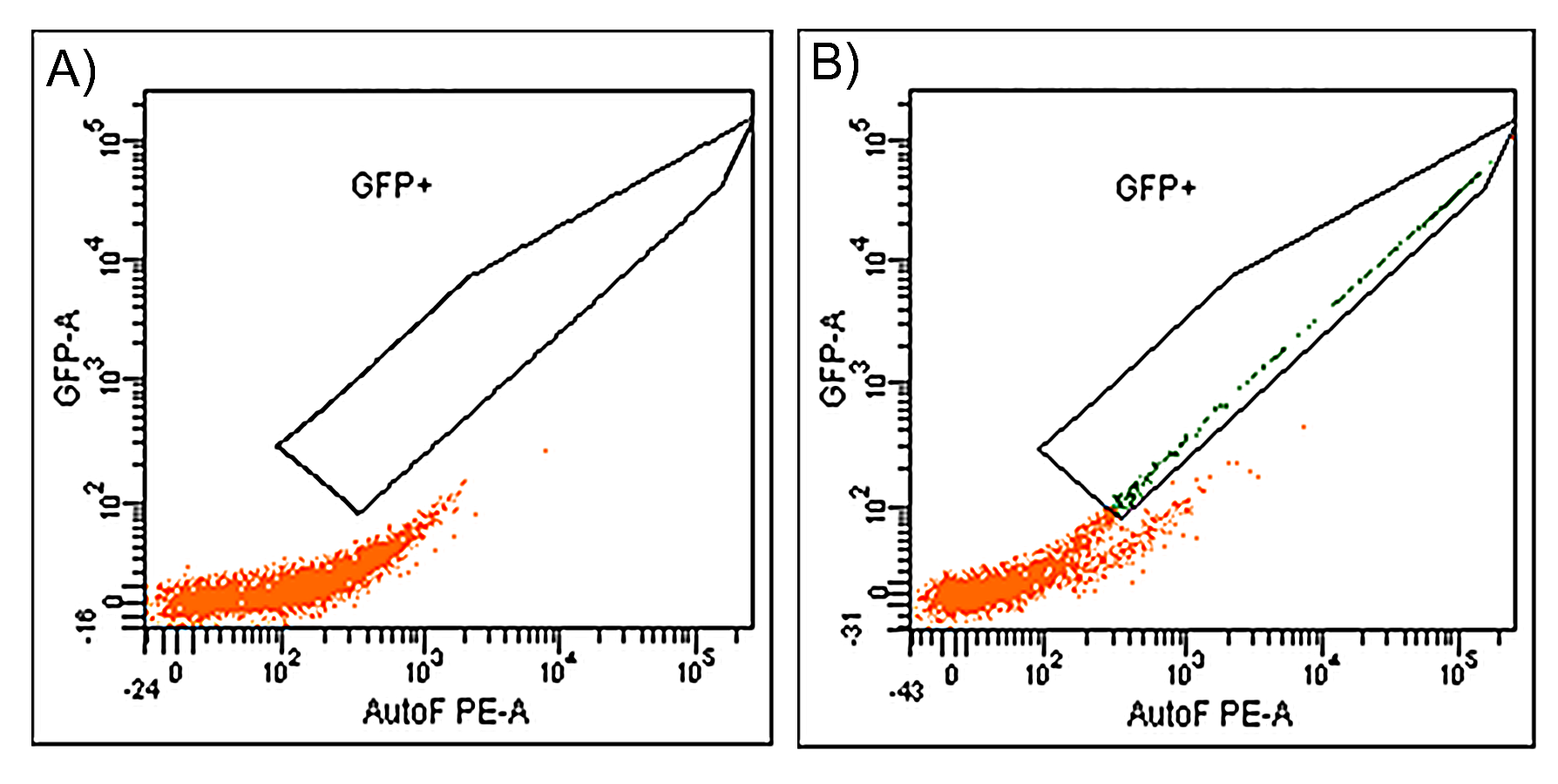

Supplement: Figure S2 — FACS of cells from non-transgenic control (Panel A), and MafB-GFP transgenic (Panel B) mice. The cells marked in green in Panel B are the GFP positive cells that were collected. (TIF) [file pone.0024640.s002.tif]
